# COVID-19: Against a Lockdown Approach

**DOI:** 10.1007/s41649-020-00154-y

**Published:** 2020-11-25

**Authors:** Steven R. Kraaijeveld

**Affiliations:** Wageningen University & Research, Wageningen, Netherlands

**Keywords:** COVID-19, Lockdown, Altruism, Freedom, Justice, Public health ethics

## Abstract

Governments around the world have faced the challenge of how to respond to the recent outbreak of a novel coronavirus disease (COVID-19). Some have reacted by greatly restricting the freedom of citizens, while others have opted for less drastic policies. In this paper, I draw a parallel with vaccination ethics to conceptualize two distinct approaches to COVID-19 that I call altruistic and lockdown. Given that the individual measures necessary to limit the spread of the virus can in principle be achieved voluntarily as well as through enforcement, the question arises of how much freedom governments ought to give citizens to adopt the required measures. I argue that an altruistic approach is preferable on moral grounds: it preserves important citizen freedoms, avoids a number of potential injustices, and gives people a much-needed sense of meaning in precarious times.

“The world can understand well enough the process of perishing for want of food: perhaps few persons can enter into or follow out that of going mad from solitary confinement.”

Charlotte Brontë, *Villette*, 323

## Introduction

The recent outbreak of a novel coronavirus disease (COVID-19) caused by the severe acute respiratory syndrome coronavirus 2 (SARS-CoV-2) was classified as a pandemic by the World Health Organization (WHO) on 11 March 2020. Given the unavailability of either a vaccine or a cure in the aftermath of the outbreak, governments around the world have faced the challenge of how best to respond so as to curb the spread of the virus. Since little is currently known about COVID-19, no single course of action has been unequivocally recommended by epidemiologists, virologists, and other experts. The Chinese government responded to the outbreak in the city of Wuhan, where the virus first appeared in December 2019, by practically shutting down life in the city. The implementation of a lockdown of this kind, which has subsequently been followed by other nations—most notably Italy, which witnessed the first major outbreak of the novel coronavirus in Europe—has been the subject of much discussion in the media and elsewhere. The focus, however, has been more on whether or not a lockdown is (likely to be) effective, than on the ethical issues that such a severe and far-reaching response raises.

In this paper, I want to examine the morality of a lockdown response to COVID-19. In order to do so, I draw a parallel to recent work on vaccination ethics, where the goal of protecting vulnerable third parties through vaccination has been argued to be achievable, at least in principle, either by leaving people free to vaccinate for the sake of others or by taking the decision out of their hands and enforcing it in some way. I conceptualize two roughly analogous approaches to the COVID-19 pandemic: an altruistic approach and a lockdown approach.^1^ The fundamental difference between the approaches is whether or not people are left at least some freedom to take upon themselves the necessary measures in light of the pandemic. A lockdown approach permits freedom of movement outside of the home only for what are judged to be the most strictly necessary activities, whereas an altruistic approach preserves at least some freedoms beyond essential undertakings.^2^

At least in principle, an altruistic approach is able to achieve the goals of an enforced lockdown. According to a recent modeling study, for instance, individual adoption of handwashing, mask-wearing, and social distancing can be an effective strategy to mitigate COVID-19; in fact, self-imposed measures were found to be able to prevent a large epidemic if efficacy exceeded 50% (Teslya et al. 2020).^3^ Assuming that the aim of COVID-19 measures is to drastically reduce the spread of the virus, or what has come to be known as *flattening the curve*, then what is needed is for people to follow the requisite procedures that will lead to the desired outcomes (i.e., self-isolating when infected, careful attention to personal hygiene, social distancing,^4^ and so on), which can in principle be achieved freely as well as through enforcement. Flattening the curve refers to community isolation measures, which, over time and compared to no intervention, are meant to slow the acceleration of new cases, to reduce the peak number of cases, and to decrease demands on hospitals and other health care infrastructure (Specktor 2020). Another way in which this has been formulated is in terms of *R*_0_, the basic reproduction number (or ratio) of the virus. *R*_0_ represents “the number of cases that are expected to occur on average in a homogeneous population as a result of infection by a single individual, when the population is susceptible at the start of an epidemic, before widespread immunity starts to develop and before any attempt has been made at immunization”; if *R*_0_ is greater than 1, the virus will spread exponentially, whereas if *R*_0_ is less than 1, the virus will spread more slowly and gradually die out (Aronson et al. 2020). Two crucial aims here are to minimize morbidity and mortality among the most vulnerable members of society and to ensure that health care systems are not overwhelmed by the number of cases requiring critical care.

To this end, adopting the necessary measures means that people will have to take on burdens—some more substantial than others. Additionally, in most cases, those burdens will have to be borne by people not primarily for their own sake, but for the sake of others. This is because the majority of citizens will not be members of the group of people who are most vulnerable (e.g., the elderly, the chronically ill, the immunosuppressed). An appeal to self-interest here is unlikely to provide sufficient motivation to take the appropriate measures. Given the goal of preventing the spread of the virus as much as possible, especially in order to protect the most vulnerable people, an important question thus emerges: To what extent ought governments to allow citizens the freedom to take upon themselves the necessary measures in response to COVID-19? The juxtaposition of lockdown and altruistic approaches as normative strategies provides the framework from which I will address this question.

I proceed as follows. First, I develop the analogy to vaccination approaches in order to show their usefulness for a discussion about the morality of government approaches to COVID-19. Second, I describe the two approaches—lockdown and altruistic—in turn, providing examples from nations that have implemented one or the other. Finally, I make a case for the moral value of taking an altruistic as opposed to a lockdown approach, focusing on the domains of freedom and justice.

Ultimately, I argue that an altruistic approach is morally preferable because it conserves some important freedoms and at least partly avoids injustices and harms associated with a lockdown approach.^5^ If an altruistic approach is found to insufficiently serve the public health goal of curbing the spread of the virus and protecting vulnerable members of society—if people do not take personal responsibility and fail to heed the call to take appropriate measures to flatten the curve—then the move toward a lockdown approach may appear warranted, perhaps even necessary. Nevertheless, the moral problems raised by a lockdown approach should neither be ignored nor downplayed, and it will still be better to introduce more stringent measures into a basically altruistic approach than to resort to locking down. Even if a lockdown is effective in reducing the spread of the virus, there are still reasons to favor an altruistic approach on moral grounds.

## Approaches to COVID-19

With the ultimate goal of limiting interpersonal contact as much as possible, especially in order to prevent vulnerable members of society from contracting the virus, the situation presented by the spread of COVID-19 parallels a dilemma that I have previously identified in relation to people vaccinating for the sake of others (Kraaijeveld 2020). What could—or should—governments do in order to increase the uptake of vaccines that do not necessarily benefit most those who would take them, but which would contribute to protecting vulnerable members of society? On the one hand, governments could rely on people to realize the importance of vaccinating (e.g., against influenza) in order to help protect vulnerable others (e.g., the elderly or immunocompromised)—even if for many people, it is the case that they personally do not stand to benefit most from the vaccine (e.g., if they are healthy young people). On the other hand, governments could decide not to rely on people’s other-regarding motives in these matters. This becomes a more pressing issue when vaccination uptake is too low to establish or maintain herd immunity—that is, when people, left to their own inclinations, are not vaccinating sufficiently on the whole to achieve the public health goal of protecting vulnerable people.^6^ Governments could then opt for a more proactive approach to vaccination, in order to explicitly increase vaccine coverage. This could be achieved through a range of methods, including more coercive measures (e.g., fines or exclusion from certain activities) and even compulsion. I have called an approach that leaves people free to decide to vaccinate for the sake of others an altruistic approach, while a more hands-on option, where governments are proactively involved in the decisions of citizens, I have called an indirect approach (Kraaijeveld 2020). In drawing a parallel to COVID-19, I will keep the term “altruistic” for the first kind of approach, while, for the sake of clarity, I will call the second kind “lockdown.”

These ideas from vaccination ethics will thus form the conceptual basis of the approaches that I describe in the following sections. Of course, once a vaccine for COVID-19 is found, this dilemma will readily present itself in relation to vaccines again. For, in order to protect those who are likely to suffer the most serious complications from COVID-19, a large number of those who are unlikely to suffer as much will likely need to be vaccinated (e.g., a sufficient number of younger people in order to establish herd immunity for those who cannot become vaccinated—like the immunosuppressed). This is an important subject for discussion, but one that I will not pursue here.^7^

The dynamic that underlies the considerations behind vaccination and the potential approaches to vaccinating for the sake of third parties to which they give rise thus translates to the current situation, where governments try to decide how best to respond to the COVID-19 pandemic. However, instead of having to decide how to regulate vaccination so as to ensure that more vulnerable people are protected by those less so, the dilemma now centers on how to regulate the adoption of relevant curve-flattening measures toward the same end. In the face of COVID-19, how should governments act in order to safeguard society’s most vulnerable populations and maintain the functioning of health care systems?

I want to clarify that I do not wish to give the impression that there have been, or that there possibly are, only two ways in which governments could respond to the pandemic. The two approaches will, to some extent, serve as ideal types. That they should do so makes sense; although I will provide empirical examples to give real-world content to the approaches, my main purpose in conceptualizing them is normative, namely to provide an answer to the question of whether a lockdown approach is morally justified—and therefore should or should not be taken by governments—in light of an alternative, altruistic approach.

Within the larger scheme of things, what one finds is that the question of what governments ought to do to halt the spread of the novel coronavirus has concentrated on whether or not they ought to enforce a complete lockdown like China did in the city of Wuhan, or whether they should instead allow citizens to retain at least some degree of freedom of movement, decision-making about their activities, and so on. It is this question concerning the freedom of citizens—especially the freedom to engage in at least some non-essential activities—that I wish to capture through my discussion of the different approaches.

### Lockdown Approach

Perhaps the most conspicuous approach to the COVID-19 pandemic has been the locking down of cities and even entire countries. The precedent of shutting down virtually all public life and of people being largely confined to their homes was set by Chinese authorities in the city of Wuhan. In Europe, Italy followed suit after experiencing the first major outbreak on the continent. Since then, a number of other countries around the world have opted to lock down. A lockdown, also known as a stay-at-home order, generally disallows all but the most essential activities for the general public (e.g., going to the supermarket, pharmacy, hospital). A great many aspects of regular public life will be affected, even while the operations of vital work and services will often be maintained. Public (and even private) modes of transportation will frequently be halted or reduced in numbers and/or operating hours, while restaurants, cafés, bars, shops, hairdressers, gyms, and numerous other places of public entertainment and services will frequently be closed. For my account, the shutting down of these public places and activities is not decisive in distinguishing the two approaches. The crucial element is whether citizens are free to stay home and to take at least some of the required COVID-19 measures upon themselves, or whether this is enforced.^8^ An altruistic approach can be compatible, for instance, with the closing down of most public places, as long as citizens are still free to leave their homes for some non-essential activities. On the other hand, it appears unlikely that a lockdown approach would not entail much of public life grinding to a halt, given that citizens have to remain home in any case.

Importantly, a lockdown has to be enforced through state power in order to be effective, so that certain acts will become criminalized. Fines will be introduced, and the threat of detainment, arrest, and in some cases even jailtime will be utilized to ensure that citizens do not flout the terms of the lockdown (whatever these may be). In this way, a lockdown approach occupies an extreme point on a spectrum where on the other end lies a laissez-faire policy that allows people to do exactly as they otherwise would (i.e., before COVID-19).

The following examples are not meant to provide a complete list of all the countries that have so far enforced a lockdown, nor is it supposed to be exhaustive of all aspects of public and private life that have been affected. I focus on three particular countries that have taken lockdown approaches, in respective chronological order: China (specifically Wuhan), Italy, and France. My purpose in doing so is to illustrate the lockdown approach and to provide a general sense of what it involves.

One report describes the situation in Wuhan after the lockdown as follows. “The streets … are eerily quiet. The city of 11 million people, the center of the coronavirus outbreak, has been locked down since 23 January,^9^ with all public transport, flights and trains suspended. ‘You pretty much don’t see anybody outside,’ says a man who lives in Wuhan… Private vehicles are banned in the downtown area. Highways are shut so residents aren’t able to leave the city” (Lu 2020, 7).

A similar picture emerges in Italy, which was the first European country to implement unprecedented lockdown measures so as “to restrict citizens’ mobility and try to contain the COVID-19 epidemic, rapidly escalating to more aggressive interventions to reduce social mixing and interrupt transmission chains,” through a range of policies “from school closure, advice against traveling or even banning non-authorized trips to and from areas with sustained transmission, university closure, ban of large-scale and public events, and then of any social gatherings, closure of museums, increasing restrictions on the opening hours of restaurants and bars, and encouraging or mandating smart/remote working whenever possible,” with nearly every day seeing “new and stricter policies … in an increasing number of Italian provinces” until finally, on March 10, “the whole country [was] under lockdown” (Pepe et al. 2020, 2).

In France, a lockdown was also instated. It became official on 17 March, and meant that “all non-essential outings [were] outlawed and [could] draw a fine of up to €135 ($148)” (Regan et al. 2020).

The upshot of these measures has been that, unless people have something justifiably urgent to do—and activities often do have to be justified, for instance by having to carry a document that indicates one’s reason for leaving home—people must remain inside their homes, at the threat of punishment.^10^ That is, the behavior that is needed to flatten the curve (i.e., social distancing, self-isolation, and so on) becomes enforced. In this way, people are not, or no longer, free to act as they see fit given the situation. To clarify: people under lockdown can violate lockdown rules, thus exercising their agency to some extent. They are still free to not follow the lockdown rules, even though this will likely come at a significant cost (e.g., through fines or even arrest). They can still choose to accept whatever consequences are at stake. However, under a lockdown, people are no longer free to decide to follow the measures required to flatten the curve for the sake of other people. There is no real choice to do the right thing, when doing the right thing is enforced—when not doing the right thing means that you will be punished. This is an important point, for reasons that I will discuss in more detail later.

### Altruistic Approach

Whereas a lockdown precludes all non-essential activities, an altruistic approach can in principle achieve the same goals of flattening the curve, while letting citizens keep at least some of their regular (i.e., pre-pandemic) freedoms. The term “altruistic” here refers not to the motives of the governments that would select such an approach—the approach *itself* is not altruistic—but to the space that governments leave citizens to behave in other-regarding ways that are necessary to prevent the spread of the virus. A different way of formulating it is that under a lockdown approach, citizens are compelled to act in ways that will collectively protect vulnerable others, while an altruistic approach allows citizens at least some freedom toward that end. This is an important difference between the two approaches, which is clearly relevant for any justification of the approaches from a public health ethics perspective.

When one speaks of altruism, the question of what exactly it means quickly arises. There is much debate in the philosophical and other literature about how to best understand the concept (Scott and Seglow 2007). I do not wish to get caught up here in a discussion about definitions. My conception of altruism is minimal; it involves doing something for someone else (or for a group of others) primarily for the latter’s sake. This will often entail taking on some kind of burden, which may be more or less significant. In extreme cases, altruism may take the form of self-sacrifice, but it does not have to. Some have argued that self-sacrifice is required for an act to be truly altruistic, but I disagree.^11^ I think that doing something for others—primarily for their sake—even when this is done at the cost of only a relatively small burden, is properly understood as an altruistic act. Altruism is not necessarily heroic; if we reserve the notion only for heroic acts, then we will both find and be able to ask very little of it. More concretely, within the present discussion, altruistic behavior is exemplified by the things that people do to avoid spreading COVID-19, primarily for the sake of others (i.e., to protect vulnerable people, to lighten the burden on health care workers, and so on). Of course, self-interest will be involved. People do not wish to become ill themselves, and people want a properly functioning health care system for when they should require critical care. Yet stressing self-interest is unlikely to be sufficiently motivating across the board, because, as I have indicated earlier, the majority of people are not going to become very ill or die from the virus. Low-risk groups, like healthy teenagers, quite simply do not face the same stakes as the elderly or the chronically ill do when they are exposed to COVID-19. These groups of low-risk and otherwise mobile and active people would therefore stay home primarily for the sake of others.

While a lockdown approach tends to be categorical (either a lockdown is in place or it is not), an altruistic approach can vary in terms of how many restrictions are introduced—as long as some basic non-essential freedom-preserving activities are retained. It allows more variation and tweaking in terms of specific policies. A lockdown approach does not have this kind of leeway; it is the all in the all-or-nothing approach, as has been demonstrated by countries like China and Italy in their respective lockdowns. Importantly, an altruistic approach does not mean that everything remains as it was before the outbreak of the virus; the guiding assumption in light of COVID-19 has been that governments do need to take some kind of action to curb the spread of the virus. An altruistic approach means that governments do proactively engage the public with regard to the importance of taking measures to flatten the curve, so as to protect vulnerable people and health care systems.^12^ Knowledge about the coronavirus and about the measures that can be taken against its spread needs to be disseminated among the public. In fact, for the approach to be properly altruistic in my sense of the term, and not simply a hands-off approach, governments have to stress precisely what citizens could and ought to do in order to flatten the curve and to help one another get through the pandemic.

When an altruistic approach is adopted, citizens are left free in some ways, then, to act responsibly. This remaining freedom could be something as relatively small (yet still significant) as being allowed to go outside for a leisurely stroll whenever one wants, rather than merely to perform a narrowly defined goal-directed activity, like going to the supermarket or pharmacy. These still-allowed activities can be paired with suitable guidelines, like making sure to keep an appropriate amount of distance from other people (e.g., the 1.5-meter rule). Again, the crucial point is that people are still allowed to do things in public spaces that are not very narrowly defined as essential.

The altruistic approach maps onto some policies that countries have already taken in response to COVID-19. In the Netherlands, for example, the National Institute for Public Health and the Environment (RIVM) has formulated three potential approaches to combat COVID-19 as part of their advice to the Dutch House of Representatives. These approaches are as follows: (1) no intervention, (2) maximum control, and (3) lockdown.^13^ The approaches that I have described roughly correspond to the latter two, with a lockdown being equivalent to my conception of it, and a “maximum control” approach resembling an altruistic approach in the demands that it makes of citizens and the freedom that it leaves them. I do not describe taking no action (no intervention) as an approach, because I think that it is clear that some intervention is required by governments of countries where infections have appeared. A discussion of the morality of taking no action whatsoever would seem to me to be rather short: it is the wrong approach. Even in a country like Sweden, which has taken a relatively hands-off approach to COVID-19 (leaving most public places open), the government has nevertheless been proactive with regard to communicating the need for social distancing. This highlights the need for governments to robustly inform and engage with the public when opting for an altruistic approach.

At the time of writing, the Netherlands has decided against a lockdown and has opted for maximum control or, in the current terminology, an altruistic as opposed to a lockdown approach.^14^ The dual goal of this approach is to protect vulnerable groups of people and to maintain the integrity of the health care system, which it aims to accomplish without locking down.

Under an altruistic approach, governments should stress the importance of solidarity and of helping others, of the need to make sacrifices for the greater good, of taking personal responsibility for the necessary measures to protect oneself and others against the virus, and so on. Numerous means of persuasion and non-coercive measures to motivate citizens to act so as to flatten the curve can and should be employed. Such means have, in fact, been used widely and creatively in places like the Netherlands. Government and health officials have taken this line by publicly emphasizing the need for solidarity and by encouraging people to act responsibly, and through television commercials and social media have underscored the importance of staying at home and protecting others. In this way, an attempt was made to offer people (1) knowledge of what needs to be done (e.g., stay home as much as possible), (2) an understanding of why this is necessary, with an emphasis on altruism and solidarity (i.e., to help flatten the curve), and (3) a sense of personal responsibility to make sure that one adheres to the necessary measures and implements the required changes.

Of course, leaving people free to decide how to act can result in the desired behavior (i.e., altruism and solidarity), but it may also lead to undesired behavior (i.e., selfishness and disregard for public health measures). This is why it is not enough for a government to do nothing—there needs to be a proactive, guiding approach that makes it very clear what is being asked of people and why it matters that they pay heed to the advice. Desired norms should be accentuated, and, in all of this, relevant insights from social psychology and related disciplines can and should be utilized (cf. Van Bavel et al. 2020).

In Sweden, a relatively relaxed approach to COVID-19 has been taken, as schools, gyms, bars, and restaurants have been left open throughout the crisis to date. Nevertheless, the government has urged citizens to behave responsibly and to follow the proper social distancing guidelines (Rolander 2020). Sweden’s approach is considered paradigmatic for its lack of stringent measures. While there is some evidence that the Swedish COVID-19 approach was able to “achieve results highly similar to late-onset stringent mandates” (Kamerlin and Kasson 2020), there is also evidence that the relatively laissez-faire approach ultimately resulted in a significant increase in mortality especially among the vulnerable (Habib 2020). More data is required to ascertain the specific consequences of different approaches. For now, however, the Swedish case at least suggests that much can be achieved by means of voluntary measures (Kavaliunas et al. 2020).

The paths taken by the Netherlands and Sweden might appear very different if one focuses on public spaces, because in the Netherlands most restaurants and places of entertainment have been shut down in response to the coronavirus. However, my conception of an altruistic approach unites the two cases: while there has been more loss of freedom in the Netherlands compared to Sweden, fundamentally the two nations have taken the same approach by not opting for a lockdown, and by allowing people freedom of movement while at the same time emphasizing the need for people to be responsible and to show solidarity with others. It is important to keep this point in mind, especially throughout the following sections, where I will argue that it is ultimately better for governments to include stricter measures within an altruistic approach than to go so far as locking down.

## Moral Issues

I will focus on a number of issues broadly within two moral domains—freedom and justice—in order to show why the lockdown approach is problematic and why an altruistic approach to COVID-19 is the morally preferable policy for governments.^15^

### Freedom

The most obvious feature of a lockdown is the restriction of freedom that it entails. This is inherent in the concept of a lockdown, which requires that people stay put where they are. When a stay-at-home order is enforced by the state, citizens are prevented from leaving their homes at their own discretion. Freedom of movement is an important human good, so that infringing on it is problematic from a moral point of view. In fact, freedom of movement is so important that Article 13 of the Universal Declaration of Human Rights states that everyone “has the right to freedom of movement … within the borders of each state” (United Nations 1948). This notion was later taken up in Article 12 of the International Covenant on Civil and Political Rights (ICCPR), which incorporates the human right to freedom of movement into treaty law, stating that “everyone lawfully within the territory of a State shall, within that territory, have the right to liberty of movement” (United Nations General Assembly 1966). When governments refuse citizens the right to move freely within the country, this constitutes a violation of their basic human rights. As such, a lockdown should not be taken lightly.

There may be some situations in which restricting freedom of movement is nonetheless justified. Article 12 of the ICCPR, for instance, includes a proviso that the right to freedom of movement may be subject to restrictions; among other things, the right can be restricted when doing so is necessary to protect public health (United Nations General Assembly 1966). A recent policy brief developed by the WHO Working Group on Ethics & SARS-CoV-2 (2020, 1) reiterates the idea that “it can be legitimate in some circumstances to introduce restrictions for the sake of protecting the health of the public”. The idea of restricting freedom in order to protect public health is linked to a widely acknowledged prima facie grounds for limiting an individual’s freedom, namely when that individual’s behavior is likely to cause harm to third parties (Holland 2015). As John Stuart Mill (1859/2003, 80) originally formulated the idea, “the only purpose for which power can be rightfully exercised over any member of a civilized community, against his will, is to prevent harm to others”. According to this line of reasoning, also known as the *harm principle*, preventing harm to others is a sufficient reason to limit the freedom of a person who might cause such harm. Relating this idea to the present situation, one might think that stopping citizens from leaving their homes prevents them from spreading the virus, and thus causing harm to others, so that limiting their freedom is justified according to the harm principle.

This conclusion is hasty. Lockdown orders are among the most extreme measures that a government can take, since they are the most restrictive of freedom, and the harm principle does not entail that, if there is some chance of harm being caused, then the severest measures are automatically justified. There needs to be some sense of proportionality, a reasonable weighing of means and ends (cf. Giubilini and Savulescu 2020). An important principle here is that of the least restrictive means, which holds that “public health measures should interfere with the autonomous freedom of individuals to the least possible or necessary extent” (Byskov 2019, 511). In relation to COVID-19 measures, harm might still be avoided under certain conditions that do not go as far in their restriction of individual freedom as a lockdown. For instance, while mass gatherings may be limited, given that the risk is high of the virus spreading and causing harm under those conditions and there are currently no alternative ways to safely accommodate mass assemblies of people, citizens might still be left free to go outside for a walk, say, simply to stretch their legs and breathe some fresh air, as long as they keep an appropriate distance from others. In this way, harm can reasonably be prevented without governments entirely encroaching upon citizens’ freedom of movement. The recent modeling study showing that individual adoption of handwashing, mask-wearing, and social distancing can prevent a large epidemic if efficacy exceeded 50% (Teslya et al. 2020) is important to consider here, as all of those measures could be taken by citizens under an altruistic approach. An altruistic approach that is sensitive to harm prevention and to flattening the curve is morally preferable to a lockdown approach because it preserves at least a basic freedom of movement.

One potential issue that is related to the issue of freedom of movement concerns the matter of privacy. One consequence of a lockdown is that it necessitates enforcement. If a government decrees that citizens are not allowed to leave their homes except for absolutely necessary activities or via special exceptions, then it follows that ways of overseeing and enforcing the decree are required, especially if violations of lockdown orders are to constitute criminal offences. That is, when citizens are not merely encouraged but positively required by law to stay home, the question of control arises. How will governments ensure that citizens actually stay home? There seems to be a slippery slope here toward increasingly invasive forms of government surveillance and privacy violations. An altruistic approach makes the need for surveillance less pressing, although even when people are allowed some freedom of movement, there may be some monitoring by governments to make sure that people adhere to whatever conditions have been stipulated.^16^ Yet the kind of control that one currently sees in Italy, for instance, where citizens have to justify to local authorities why they are leaving their homes (through documentation known as *autocertificazione*) is unthinkable under an altruistic approach. Perhaps it is not inevitable that citizens have to justify their (otherwise innocuous) movements to the state under a lockdown, but it should worry us that the lockdown approach taken by a democratic country like Italy has resulted in such a state of affairs.

There are also more pragmatic reasons to limit citizens’ freedoms as little as possible. A lockdown is a heavy burden to bear, and it is unlikely that people will be able to keep up being shut inside their homes for a long stretch of time. Reactance (also known as “lockdown fatigue”) may very well develop to lockdown measures; in fact, there have been widely publicized protests against lockdowns in the USA as well as in a number of European countries like France and Germany. Even citizens who are initially prepared to make sacrifices may eventually tire of overly strict measures. Given that COVID-19 may be with us for a considerable while longer, governments have to consider carefully how best to ensure that citizens can take reasonable measures against the virus in a sustainable way, without needlessly exhausting endurance. There is some tentative evidence, based on an analysis of Google Trends, which suggests that lockdowns around the world have substantially increased the search intensity for terms like boredom, worry, and loneliness (Brodeur et al. 2020). Although this is admittedly speculative, given that there is currently no data (as far as I know), I would suggest that being allowed to go outside for leisure—and not merely for the essential tasks—will make other COVID-19 measures more bearable, and is likely to lead to less boredom, among other things, than when staying home is strictly enforced. Empirical research in this area is needed.

I have so far focused on the idea of freedom largely to argue against a lockdown approach. I want to make a final point regarding freedom here that speaks more positively to the direct moral value of an altruistic approach. There is a normative argument to be made for giving people the space to be altruistic and for allowing them to express solidarity with their fellow human beings, especially during a time of crisis. Freedom is necessary for altruism; the two concepts are intimately linked, because altruism depends on the proper kind of self-chosen motive (Seglow 2004). Differently put, if someone has no choice but to act a certain way, then the way in which one acts cannot be altruistic. Without freedom, there is no responsibility. Once a lockdown is enforced, room for altruism in this area is more or less squeezed out of society. There will be much less space, if any, for people to act based on other-regarding motives. Choosing to self-isolate for the sake of others can give meaning to one’s situation. It can make it more bearable to stay home if one knows that one is doing it by choice and for good reasons. I think that this is an important but underappreciated point.

There is empirical support for this idea. In a series of three studies, Klein (2016) found that people who engaged in prosocial behaviors (like volunteering or spending money to benefit others) subsequently reported experiencing a greater sense of meaning and purpose in their lives. Seeing one’s life as meaningful is crucial to human existence; it is associated with greater longevity (Krause 2009), better physical health (Taylor et al. 2000; Hooker et al. 2018), and reduced depression, anxiety, and overall psychological distress (Debats et al. 1993). Prosocial behavior has also been linked to greater psychological flourishing (Nelson et al. 2016) as well as an increase in well-being and vitality—even in the absence of direct contact with a beneficiary (Martela and Ryan 2016). Altruistic attitudes, volunteering, and informal helping behaviors were also found to uniquely contribute to the maintenance of life satisfaction, positive affect, and psychological well-being among retirement community member dwellers (Kahana et al. 2013). Although none of this research was conducted during the COVID-19 pandemic or a similar state of affairs, it stands to reason that people who remain at home for the sake of others will experience a greater sense of meaning and purpose compared to those whose staying at home is strictly enforced by the state. The actions of people who take measures upon themselves, because they know that they are thereby helping others, can experience a sense of meaning that those who are doing the same at the risk of punishment cannot. This line of reasoning again suggests the importance for governments of actively promoting and fostering the kind of prosocial behaviors that are not only necessary for a collective response to COVID-19 but which also stand to offer people a much-needed sense of meaning in existentially uncertain times.

Respect for autonomy—for allowing people to make their own decisions and decide how to live their lives—is an important moral principle in and of itself (Beauchamp and Childress 2012). It is a principle that is seriously challenged by a lockdown approach. I suggest that, as much as it is problematic for freedom and autonomy to be undermined by a lockdown, it is also especially good to offer people a window through which to act for the sake of others. It can give people a sense of meaning and purpose, which is always good but particularly so during a time of global crisis. This is another reason why governments ought to favor an altruistic approach.

Having discussed several matters concerning freedom, I will now address some issues related to justice.

### Justice

Only in hindsight will we be able to more fully assess the negative ramifications of the COVID-19 pandemic. As things stand, it is clear that the virus is having a detrimental impact on the lives of a great many people around the world, even if only indirectly—through the global economic consequences of the crisis, for instance. In countries where far-reaching measures have been taken in response to the pandemic, citizens have to bear not only the burdens of the virus itself but also (and perhaps especially) the particular burdens of the imposed measures. Generally speaking, the stricter the measures, the greater will their impact be on people’s everyday lives.

I want to focus here on one area of justice, namely the fair distribution of the burdens associated with COVID-19 measures. One might think that a general lockdown is eminently democratic: after all, everyone—rich and poor, young and old—has to stay at home. However, this is far from the case. When citizens are confined to their homes, there are reasons to consider it very unlikely that the burdens of a lockdown will be fairly distributed among the population. More specifically, there are at least two ways in which a lockdown approach can be unjust, in the sense that the burdens of the measures will be experienced disproportionately more acutely by some individuals and groups than by others.

First, there is what might be called the *unequal home conditions* argument. Home conditions for citizens are bound to vary greatly, so that some people are much more likely to suffer from having to stay home than others. For instance, while having to remain at home for long periods of time is tough on anyone, it is bound to be much more burdensome for people of lower socioeconomic status than for those of higher economic status. The very rich will tend to have access to comfortable accommodations, plenty of living space in which to spend their time, gardens for fresh air and exercise, and so on. At the same time, those who are less well-off will often find themselves confined to small apartments, perhaps even single rooms, with little chance of getting fresh air. After all, especially in large industrialized cities, gardens and balconies are a luxury. Those who are more well-off are likely to be able to live comfortably and independently for a significant stretch of time, having the means to afford all sorts of deliveries of goods, online means of entertainment, and so on. The least well-off, on the other hand, are often dependent on others in important ways: on the kindness of friends and family for help getting by, on food banks, and so on. Continued access to these important services by others will not always be guaranteed.

Other than socioeconomic status, forced isolation will be especially difficult for other vulnerable members of society. For instance, there is a serious risk that people with psychological problems (e.g., those suffering from depression or anxiety disorders) will suffer disproportionately from enforced isolation. There is evidence that, compared to 2018 numbers, US adults at the height of the pandemic in April 2020 were eight times more likely to fit the criteria for serious mental illness (27.7% vs. 3.4%), with especially pronounced differences among younger adults and those with children (Twenge and Joiner 2020). These statistics are not specific to lockdown conditions or to vulnerable populations, but they do suggest that, if the general population is experiencing increased mental disturbance, then those already at risk are likely to be especially stricken. A survey assessing mental health outcomes in the Italian general population three to four weeks into national lockdown measures against COVID-19 found high rates of negative mental health outcomes; among 18,147 individuals who completed the questionnaire, endorsement rates for post-traumatic stress symptoms (PTSS) were 6604 (37%), for depression, 3084 (17.3%), for anxiety, 3700 (20.8%), for insomnia, 1301 (7.3%), for high perceived stress, 3895 (22.9%), and for adjustment disorder, 4092 (22.9%) (Rossi et al. 2020). During a lockdown, the anxious stand to become more anxious, the loneliest even lonelier. People suffering from domestic violence, which may increase under conditions of isolation, when frustrations increase as outlets for violence dwindle, may also be at greater risk of being harmed under lockdown conditions, when they may quite literally have nowhere to go. Part of the logic of a lockdown is undermined by these considerations: while a lockdown is meant to protect the most vulnerable members of society, it ends up disproportionally hurting its most vulnerable populations. That it should do so is unjust.

Second, there is what might be called the *unequal geographical disease burden* argument, which is related to the proportionality principle and that of the least restrictive means. The incidence and impact of COVID-19 is unlikely to be (even roughly) equally distributed across geographical regions within a given country. If the prima facie justification of a lockdown is that it is immediately necessary in order to prevent the spread of the virus and to flatten the curve, then, assuming this justification to hold, it will apply most readily to areas where there is a significant amount of infection that needs to be curbed. It may not be apt, however, for areas where the incidence and rate of infection is very low. For instance, the decision by the Italian government to lock down the entire country in response to COVID-19 might, at least on the surface, appear to be justified from the perspective of parts of the country that became coronavirus hotspots, like the region of Lombardy. While my arguments suggest that even in those areas, a lockdown is morally problematic, there is an additional argument to be made that it might be particularly unjust if such stringent lockdown measures are also enforced in other regions, like Molise or Basilicata, where the relative impact of the virus is much smaller.

Of course, there may be reasons to introduce stricter measures in these areas, too (e.g., to keep infection rates low). Yet, to also enforce a lockdown in little-affected areas, especially if they are far removed from coronavirus hotspots, still appears to require more justification than it does in those areas where the virus is rampant. The Chinese government did not lock down the entire nation in response to the pandemic; they enforced lockdown measures first in the city of Wuhan, and later in other, more or less circumscribed areas that saw outbreaks of the virus (like in the province of Jilin). Had the government enforced measures as strict as those in Wuhan for the entire nation, one might rightfully have questioned whether this were a just policy, on the grounds that it would seriously encroach on people’s freedom while other measures, more respecting of liberty and autonomy, could have been maintained.

This argument admittedly leaves room for local lockdowns in areas greatly affected by the virus, but I have argued that there are other reasons to think that a lockdown is unjust. The unequal geographical disease burden argument suggests that a lockdown approach taken by a government for an entire nation in order to tackle the virus within a particular region can be unjust in another way.

## Conclusion

Governments around the world have needed to respond quickly to the COVID-19 pandemic. If the public health goal is to protect vulnerable people from contracting the novel coronavirus and to prevent health care systems from being overrun with cases—that is, if we all need to contribute to flattening the curve—then this poses a dilemma similar to one that is found in vaccination ethics. The dilemma centers on the question of whether governments should leave room for people’s altruistic inclinations, or whether they ought to bypass these and enforce the required measures in some ways. I have conceptualized two potential approaches to COVID-19, and I have argued that an altruistic approach is morally preferable to a lockdown approach. An altruistic approach maintains important citizen freedoms, is more respectful of personal autonomy, is less prone to result in immediate privacy violations, and avoids a number of injustices. Importantly, this approach also leaves citizens a greater sense of individual responsibility and freedom to act on altruistic inclinations, thus allowing them to give meaning to their actions and their lives during a time when despair over lack of control is a real concern.

All in all, then, governments ought to favor an altruistic approach on moral grounds. Given that the approaches are ways of responding to a crisis, and are meant to meet crucial and ongoing public health goals, there should be room for re-assessment based on empirical feedback. Should a more permissive altruistic approach that lets people almost entirely free to take action to flatten the curve fail to result in the necessary responses, then stricter measures may yet be justified. However, even then, these measures ought to be introduced within an approach that is in principle altruistic, and which does not go as far as confining people to their homes all together.

While the different approaches that I have described immediately apply to the present situation, both in guiding decisions about how to govern and in determining the morality of policies already taken, the distinction between altruistic and lockdown approaches will also be relevant to future outbreaks. One hopes, of course, that the question of how much freedom governments ought to leave citizens in the face of pandemics never arises again, but it is likely that even after COVID-19 has become a painful memory, the way in which governments handle such crises will remain a subject of much concern—including moral.

## Footnotes

1 I introduce them to address the recent COVID-19 pandemic, but the approaches—and the moral issues that they raise—may also be usefully applied to other disease outbreaks, both past and future.

2 What constitutes an essential activity may be a matter of some interpretation and may vary between countries. It is certainly up for debate, and will likely be a political decision; governments decide what citizens still need to be able to do, in which activities that may still engage, and so on.

3 It must be noted that this model included short-term government-imposed social distancing, rather than self-imposed social distancing.

4 I prefer the term “physical” to “social” distancing, because it importantly avoids connotations of reduced sociality. However, the term has stuck since it was first introduced, so I will adopt it throughout this paper.

5 It may appear straightforward that an approach that preserves freedom is preferable to one that does not, but governments might choose (and, I think, some have chosen) a lockdown without necessarily (1) realizing fully the moral costs of doing so and (2) considering investing instead in the alternative that I outline in this paper. Thanks to an anonymous reviewer for pressing me on this point.

6 One might think that vulnerable people ought simply to be vaccinated, thus eliminating the risk of infection altogether. However, aside from the fact that not all vaccines offer complete protection against a disease, so that a vulnerable person who is vaccinated will additionally benefit from the presence of herd immunity (as it further decreases their chance of becoming ill), there are groups of people who cannot be vaccinated. Among those are, for instance, infants who are too young, people who are immunosuppressed (e.g., those undergoing chemotherapy), and people who are allergic to particular vaccines.

7 For some relevant literature in this area, however, see Bambery et al. (2018) on vaccinating children—rather than healthcare professionals and individuals in high-risk groups—against influenza; and see Giubilini et al. (2020) on vaccinating the young in order to protect the old in the case of a COVID-19 vaccine.

8 An additional element of the altruistic approach is that governments stress the importance of citizens taking the necessary measures, even if these are not enforced. I will discuss this in more detail later.

9 All dates in this section refer to the year 2020.

10 This is assuming that people have homes. The question of how—and perhaps especially how not—to treat homeless people during the COVID-19 pandemic is a very important one, which nevertheless lies outside of the scope of this paper.

11 For an overview of positions on altruism, see Kraut (2020).

12 In a manner similar to how governments should highlight the importance of vaccinating for others even without enforcing such vaccination (Author Self Identifying Reference).

13 For an overview (in Dutch), see: https://www.tweedekamer.nl/sites/default/files/atoms/files/20200325_briefing_coronavirus_tweede_kamer_presentatie_rivm.pdf

14 The “maximum control” approach was later called an “intelligent lockdown” by the Dutch government. Terminology matters, of course, yet for the purpose of this paper what is decisive is not what an approach is called locally, but what it means for citizens—especially with regard to the freedoms that it permits. For more information regarding the decision, see (in Dutch): https://www.tweedekamer.nl/sites/default/files/atoms/files/20200325_briefing_coronavirus_tweede_kamer_presentatie_rivm.pdf

15 I focus on these two not because they are the only areas of moral concern, but because I have to prioritize given limited space. Where relevant, I will touch briefly on other morally relevant matters (like privacy). I leave it to others to develop these and other moral issues more fully.

16 Of course, under an altruistic approach, privacy concerns are still likely to arise during the pandemic. Tracking people’s movements and health conditions may be part of public health measures against the virus, even apart from whether or not there is a lockdown (or perhaps even as a condition to prevent a lockdown). It still appears to me that, as far as controlling individual movement goes, there is reason to think that a lockdown approach stands to exacerbate privacy concerns in the manner that I have outlined. Thanks to an anonymous reviewer for raising this point.
